# Mortality reduction in patients with severe sepsis and septic shock through a comprehensive sepsis initiative

**DOI:** 10.1186/cc14031

**Published:** 2014-12-03

**Authors:** K Nguyen, L Cook, EP Greenlee

**Affiliations:** 1 Performance Improvement Department, El Camino Hospital, Mountain View, CA, USA; 2 Emergency Department, El Camino Hospital, Mountain View, CA, USA; 3 Clinical Effectiveness, El Camino Hospital, Mountain View, CA, USA

## Introduction

The Integrated Nurse Leadership Program (INLP), a regional quality improvement initiative to reduce deaths from sepsis, began in 2008. Nine participating hospitals were provided networking opportunities and training on: care of the septic patient, program and leadership development, and data collection and analysis. El Camino Hospital (ECH) applied these concepts during implementation of the early goal-directed therapy bundle as recommended by the Surviving Sepsis Campaign and the Institute for Healthcare Improvement. This paper describes the implementation process and presents data analysis from the 22-month project, focusing on severe sepsis and septic shock mortality.

## Methods

A multidisciplinary team approach guided the sepsis initiative. Lean methodologies such as: root cause analysis, process mapping, data collection and direct observation were applied. Frequent meetings with executive personnel, frontline staff and physician leaders occurred to evaluate current practices. Policies and procedures were created, including standardized screening tools and order sets for early identification and management. Training materials (modules, lectures, handouts and simulations) were developed. Extensive training occurred at all levels, with experts presenting to large groups of clinicians. Ongoing data analysis included screening tool and bundle element compliance as well as ICD-9-based mortality among patients with severe sepsis and septic shock (excluding patients <18 years old, pregnant, or designated 'Do Not Resuscitate' within 24 hours of presentation). Length of stay (LOS) data were compared with data from a large healthcare collaborate quality alliance (Premier) using US Medicare Severity Diagnosis-Related Group sepsis codes (MSDRG 870, 871, 872).

## Results

**Figure 1:**
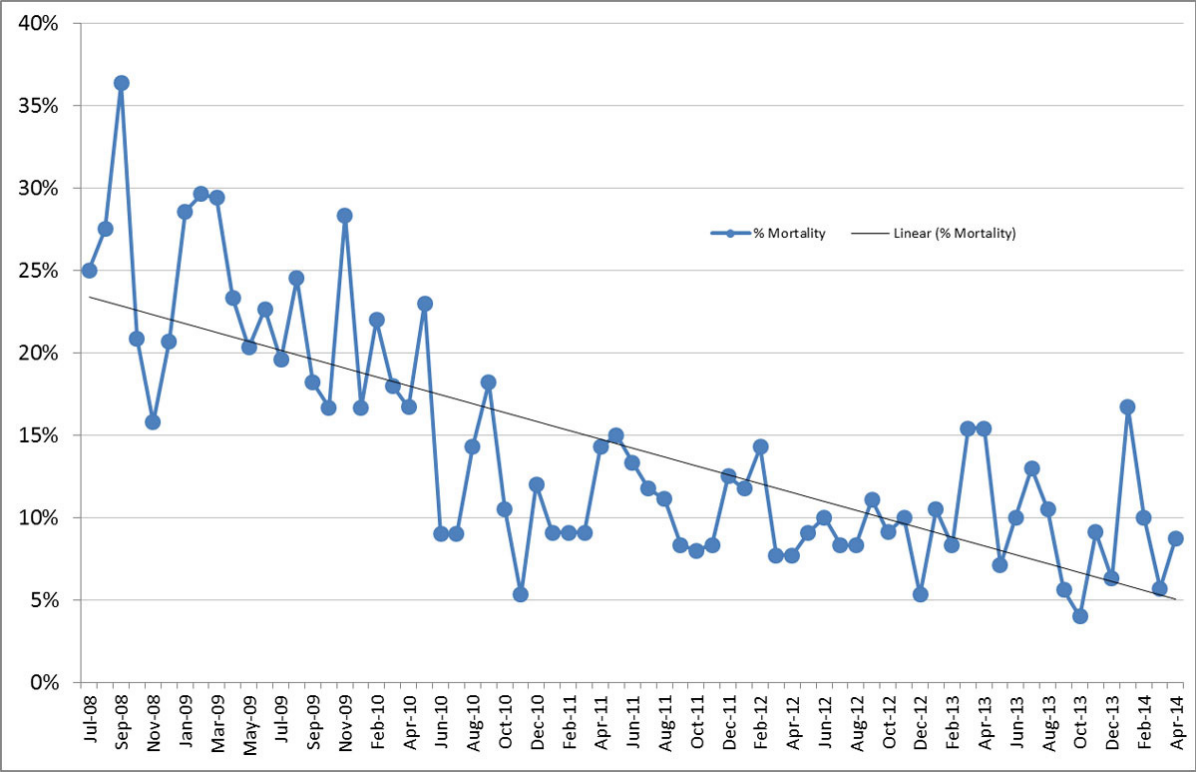
**Mortality among patients with severe sepsis and septic shock; excluding patients <18 years of age, pregnant females and patients designated 'Do Not Resuscitate' within 24 hours of presentation**.

**Figure 2:**
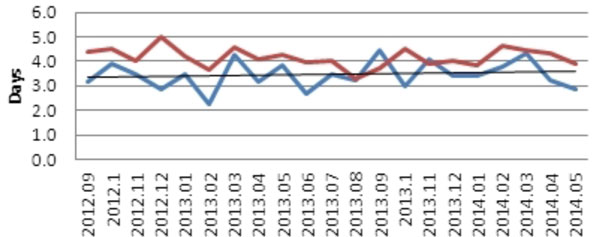
**Average length of stay for patients with sepsis codes (MSDRG 870, 871, 872), comparing El Camino Hospital to expected LOS from the Premier database**. Red line, Premier database expected LOS for sepsis patients; blue line, El Camino Hospital LOS for sepsis patients.

Prior to implementation of the program (April 2009) through April 2014, ECH achieved a relative reduction of 68% in the mortality rate among those with severe sepsis and septic shock (*P *= 0.03). This equates to 1,456 lives saved (Figure 1) [1-3]. During the initiative ECH maintained a lower than expected average LOS compared to other hospitals within the Premier database (Figure 2).

## Conclusion

The significant and sustained decrease in mortality among those with severe sepsis and septic shock was achieved through the structure and support of multisite collaboration with the INLP and robust internal operations [1,3]. The focus is now on sustainability; including key elements pertaining to accountability, affordability, compassionate care and systematic excellence [4].
